# Small Bowel Obstruction Associated With a Mesenteric Lymphatic Malformation: A Case Report

**DOI:** 10.7759/cureus.103671

**Published:** 2026-02-15

**Authors:** Masayuki Shinoda, Satoshi Inose, Homare Ito, Nobuyuki Kanai, Katsumi Kurihara

**Affiliations:** 1 Department of Surgery, Shin-Oyama City Hospital, Oyama, JPN; 2 Department of Surgery, Jichi Medical University, Shimotsuke, JPN; 3 Department of Pathology, Shin-Oyama City Hospital, Oyama, JPN

**Keywords:** case report, lymphangioma, lymphatic malformation, mesentery, small bowel obstruction

## Abstract

Mesenteric lymphatic malformations are rare benign lesions that may cause intestinal obstruction due to extraluminal compression. A 60-year-old man presented to his primary care physician with acute lower abdominal pain. Acute appendicitis was suspected based on clinical assessment, and conservative treatment was initiated. Although symptoms and laboratory findings improved, a palpable mass persisted in the right lower abdomen, and he was referred to another hospital. Further evaluation revealed small bowel obstruction, and he was transferred to our institution for further management. Contrast-enhanced abdominal computed tomography (CT) showed small bowel obstruction associated with extrinsic compression from an extraluminal mass lesion. Emergency surgery was performed on the second day after admission. Intraoperatively, multiple irregularly shaped and multilocular mass lesions of various sizes were observed in the small bowel mesentery, compressing the small intestine and resulting in obstruction. Partial resection of the small intestine including the involved lesions was performed. The postoperative course was uneventful, and the patient was discharged on postoperative day 9. Histopathological examination confirmed the diagnosis of mesenteric lymphatic malformation. Small bowel obstruction caused by extraluminal compression from a mesenteric lymphatic malformation is rare, particularly in adults. This case highlights the diagnostic challenge of extraluminal causes of bowel obstruction and supports surgical resection with complete excision as an effective treatment strategy. No recurrence or postoperative complications were observed during approximately two months of follow-up.

## Introduction

Postoperative adhesions are the most common cause of small bowel obstruction, followed by hernias and neoplastic causes. Less frequent etiologies include intestinal volvulus and other mechanical conditions [1]. Although intraluminal and adhesive causes account for the majority of cases, extraluminal compression by intra-abdominal lesions is an uncommon but important etiology. Among these, mesenteric lymphatic malformations are rare benign entities that can occasionally cause mechanical bowel obstruction. We report a case of small bowel obstruction associated with extrinsic compression from a mesenteric lymphatic malformation.

This case was previously presented at the 86th Annual Congress of the Japan Surgical Association on November 22, 2024.

## Case presentation

A 60-year-old man presented to his primary care physician with acute lower abdominal pain without associated nausea, vomiting, constipation, or diarrhea. Acute appendicitis was suspected based on the localized right lower abdominal pain and physical examination findings, and he was admitted on the same day. After five days of conservative treatment, including fasting, intravenous fluids, and antibiotics, he was discharged. Although his abdominal symptoms and blood test findings improved, a palpable mass remained in the right lower abdomen. Therefore, he was referred to another hospital and admitted for further investigation. Contrast-enhanced computed tomography (CT) revealed a bowel obstruction, which was suspected to be caused by an extraluminal mass lesion, and he was subsequently transferred to our hospital in November 2023 for further management.

Past medical history included a myocardial infarction treated with coronary stenting at age 58, as well as hypertension and hyperlipidemia. There was no particular family history, and medications included aspirin, prasugrel hydrochloride, lansoprazole, rosuvastatin, enalapril maleate, and ezetimibe. He was receiving dual antiplatelet therapy with aspirin and prasugrel hydrochloride at the time of surgery.

On physical examination at admission, the patient’s height was 159.1 cm and weight was 56.7 kg, with a body mass index of 22.4 kg/m². Vital signs were as follows: body temperature 36.3°C, heart rate 113 beats/min, blood pressure 132/95 mmHg, and oxygen saturation 97% on room air. Abdominal examination revealed tenderness, with the most intense pain localized to the right lower abdomen.

Blood tests showed no elevation of tumor markers such as carcinoembryonic antigen (CEA) and carbohydrate antigen 19-9 (CA19-9), and no significant elevation of soluble interleukin-2 receptor (sIL-2R). Mild elevations of liver enzymes were observed, but no increases in inflammatory markers such as white blood cells (WBC) and C-reactive protein (CRP). There was also no elevation in lactate.

**Figure 1:**
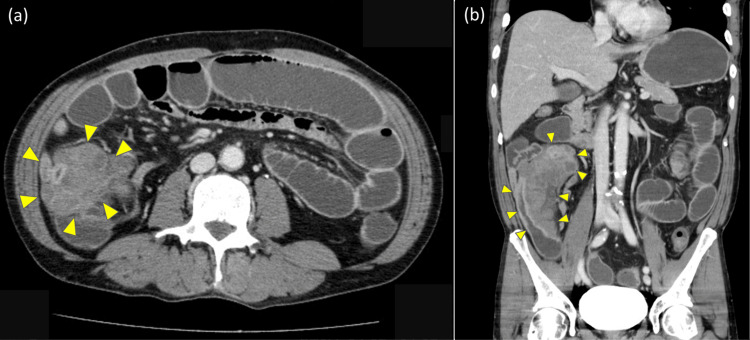
Abdominal contrast-enhanced CT findings. (a) Axial image showing dilatation of the proximal small intestine and extraluminal mass lesions adjacent to the right small intestine causing luminal stenosis. (b) Coronal image demonstrating extrinsic compression of the right small intestine by the extraluminal mass lesion. CT: computed tomography.

Abdominal contrast-enhanced CT revealed extensive dilatation of the small intestine with fluid stasis. A mass lesion was observed extraluminally adjacent to the small intestine, causing extrinsic compression in the right abdomen and resulting in severe luminal narrowing. No other obstruction, ascites, or findings suggestive of intestinal ischemia or necrosis were observed (Figure 1a, b).

**Figure 2:**
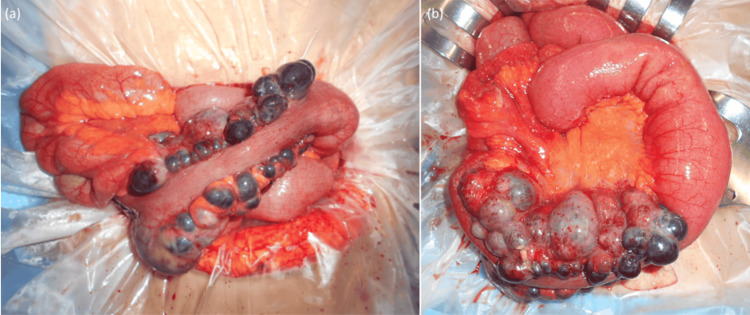
Intraoperative findings. (a) The masses originating from the mesentery are compressing the small bowel. (b) The proximal small bowel is dilated, while the distal side is collapsed.

Based on these findings, small bowel obstruction was diagnosed, and the extraluminal mass lesion was suspected to be the cause. Under general anesthesia, the patient was placed in the supine position, and laparotomy was performed via an oblique incision in the right upper abdomen. Intraoperative findings included an enlarged small bowel with a small amount of reactive ascites. Multiple mass lesions of varying sizes were identified in the small bowel mesentery compressing the small intestine, with dilation of the proximal bowel and collapse of the distal segment (Figure 2a, b).

**Figure 3:**
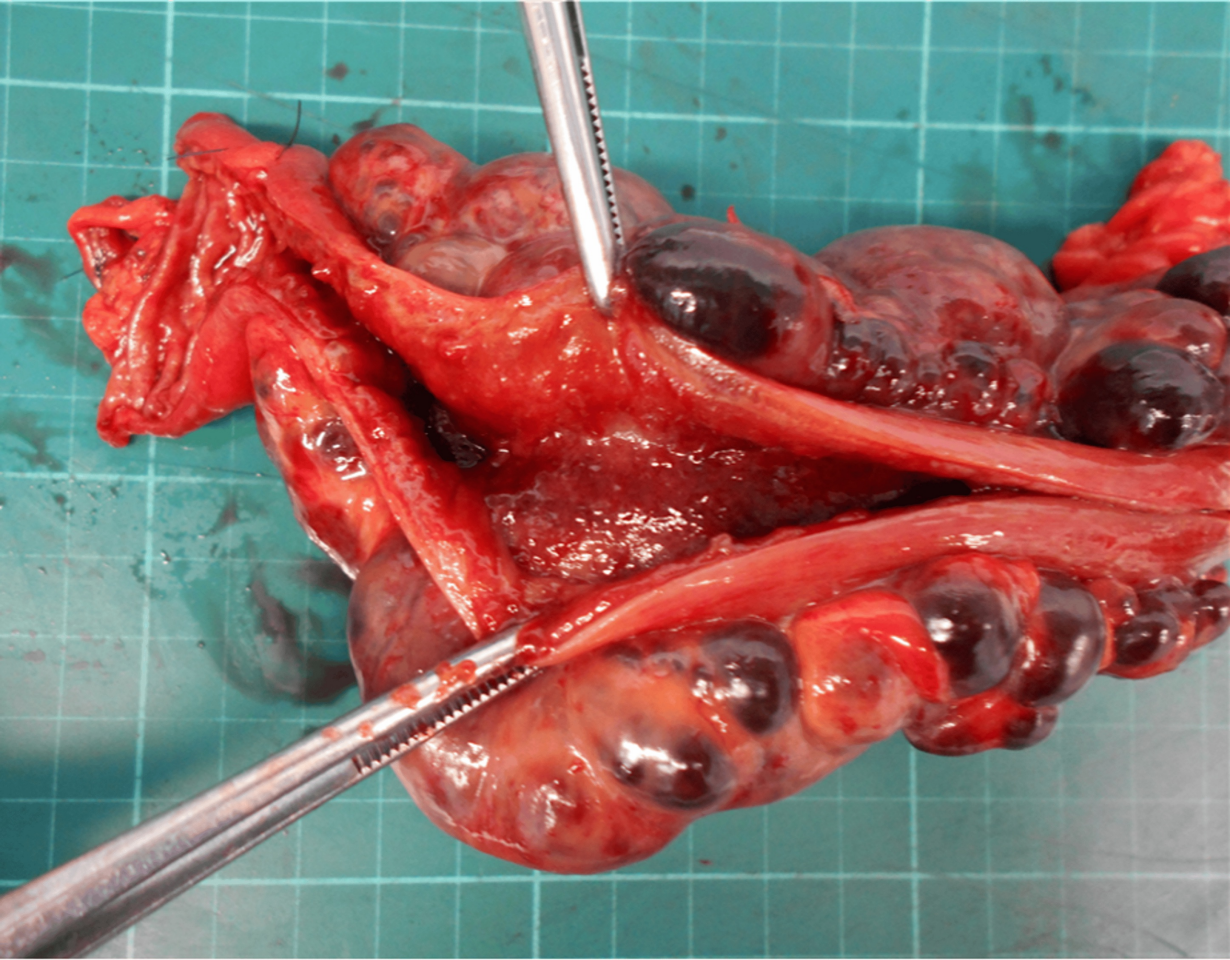
The mucosal surface of the resected small bowel. No intraluminal abnormalities were observed.

The obstructed segment was located approximately 60 cm from the terminal ileum, and no other similar lesions were identified. Based on these findings, approximately 20 cm of the small intestine, including the involved mesenteric lesion, was resected, followed by reconstruction with an anastomosis using a Gambee suture. Simple decompression without bowel resection was considered difficult due to the extent of mesenteric involvement and the close anatomical relationship between the lesion and the adjacent small bowel, resulting in fixed extraluminal compression. The operative time was 90 minutes with minimal blood loss. The resected small bowel was opened, and no intraluminal abnormalities that could potentially cause obstruction were observed (Figure 3).

**Figure 4:**
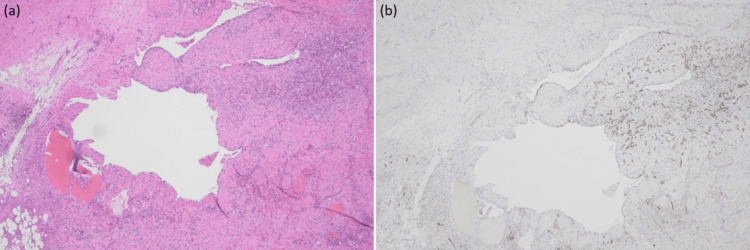
Histopathological findings. (a) Hematoxylin and eosin staining shows multiple dilated cystic spaces lined by flattened endothelial cells without atypia. (b) Immunohistochemical staining demonstrates positivity for D2-40 in the endothelial lining, supportive of lymphatic origin.

The postoperative course was uneventful, and the patient was discharged on postoperative day 9. At approximately two months of follow-up after discharge, the patient remained asymptomatic with no evidence of recurrence. Histopathological examination revealed multiple hemorrhagic and dilated cystic lesions arising from the small bowel mesentery, which compressed the adjacent small bowel. Histologically, flattened endothelial cells lined the cystic spaces, and no atypia was observed. The endothelial cells showed positive immunohistochemical staining for D2-40 (Figure 4).

To exclude other mesenchymal tumors, additional immunohistochemical staining for CD34, β-catenin, and desmin was performed. Based on these histopathological findings in conjunction with the macroscopic features, a diagnosis of mesenteric lymphatic malformation was confirmed. 

## Discussion

According to the International Society for the Study of Vascular Anomalies (ISSVA) classification, vascular anomalies are divided into vascular tumors and vascular malformations. Lymphatic malformations are classified as vascular malformations and are characterized by congenital, non-proliferative structural abnormalities of the lymphatic vessels [2]. The term lymphangioma has traditionally been used. However, lymphatic malformation is currently preferred in accordance with the ISSVA classification, reflecting its non-neoplastic biological behavior.

Lymphatic malformations are benign congenital lesions that predominantly present during childhood. However, adult cases have also been reported, including those arising in intra-abdominal locations such as the mesentery [3]. In adult cases, mesenteric lymphatic malformations have rarely been reported to cause acute abdominal complications, including small bowel obstruction, intestinal volvulus, and mesenteric ischemia [4]. Recent case reports have documented adult patients with mesenteric lymphatic malformations who developed mechanically induced small bowel obstruction and underwent surgical resection with favorable postoperative outcomes [5].

Mesenteric lymphatic malformations may be asymptomatic or present with nonspecific clinical features [6], and some cases are detected incidentally [7]. However, depending on size and location, these lesions may exert extrinsic compression or traction on the bowel, potentially leading to mechanical complications such as small bowel obstruction or intestinal volvulus [4-6]. Recent case reports in adult patients have described mesenteric lymphatic malformations associated with intestinal volvulus, which can result in small bowel obstruction, and in some cases mesenteric ischemia requiring emergency surgical intervention [4,6]. Therefore, although mesenteric lymphatic malformations are benign entities, they should be recognized as a potential cause of acute abdominal complications in adult patients.

Preoperative diagnosis of mesenteric lymphatic malformations is often challenging because their clinical symptoms are nonspecific and may be absent or mild in some cases [4,8]. In the symptomatic cases, imaging studies such as ultrasonography and contrast-enhanced CT are useful for revealing findings such as multiloculated cystic lesions and extrinsic compression of adjacent bowel loops [4,8]. However, definitive diagnosis cannot be established by imaging studies alone and requires histopathological examination [4,8]. Immunohistochemical staining, particularly positivity for D2-40, is helpful in supporting lymphatic endothelial origin and in the differential diagnosis of other cystic or mesenchymal tumors [4,7,8]. In this case, histopathological evaluation, including positive D2-40 staining, supported the mesenteric lymphatic malformation diagnosis.

Treatment strategies for mesenteric lymphatic malformations include surgical resection and conservative observation. The choice of treatment is determined based on the location and the size of the lesion, as well as the presence of symptoms [7]. Previous reports indicate that surgical resection is generally the first-line treatment for symptomatic cases, particularly those presenting with symptoms such as small bowel obstruction, volvulus, or mesenteric ischemia [4,5]. Previous case series have suggested that complete surgical resection is associated with favorable outcomes and low risk of recurrence [7]. Particularly in terms of curative potential and safety, surgical treatment is preferred in cases involving bowel compression or obstruction [4,7]. In this case, small bowel obstruction was successfully treated by segmental resection of the involved small intestine including the causative lesion, resulting in complete malformation removal and an uneventful postoperative course.

## Conclusions

We report a case of small bowel obstruction caused by extrinsic compression from a mesenteric lymphatic malformation. Contrast-enhanced CT contributes to preoperative clinical assessment, and characteristic intraoperative gross findings, such as multilocular cystic lesions in the mesentery, together with histopathological examination, confirm the diagnosis. Mesenteric lymphatic malformations should be considered in the differential diagnosis, as they can cause an acute abdomen due to bowel obstruction or volvulus. Surgical management should be tailored to each case, with consideration of complete resection of the lesion.
